# Antiapoptotic Molecule Survivin in Transplantation: Helpful or Harmful?

**DOI:** 10.1155/2018/6492034

**Published:** 2018-10-01

**Authors:** Sara Assadiasl, Mohammad Javad Mousavi, Aliakbar Amirzargar

**Affiliations:** ^1^Molecular Immunology Research Center, Tehran University of Medical Sciences, Tehran, Iran; ^2^Department of Immunology, School of Medicine, Tehran University of Medical Sciences, Tehran, Iran; ^3^Department of Hematology, Faculty of Allied Medicine, Bushehr University of Medical Sciences, Bushehr, Iran

## Abstract

Survivin, an antiapoptotic molecule from inhibitor of apoptosis protein (IAP) family, is most known for its implication in cancer as there are some efforts to apply it for diagnostic as well as therapeutic purposes in oncology. On the other hand, it could be a useful molecule to be positively targeted when trying to save tissue and promote cells viability. Since protecting the allograft from ischemia reperfusion injury and inflammation-induced damage is a considerable objective in transplantation, it is reasonable to take advantage from antiapoptotic agents like survivin in order to achieve this goal. However, survivin's potential ability to induce malignancies makes some concerns about its use in clinic. The other barrier is this molecule's involvement in lymphocytes development and proliferation which might increase the risk of graft rejection due to adaptive immune system overactivation. In this review we summarize the few studies carried out about survivin's effect on graft survival and probable advantages and disadvantages of its overexpression in transplantation.

## 1. Introduction

To keep up with the rapid surgical and pharmacological advancements in transplantation and the rising number of organ recipients, there is a need for designing fast non-invasive graft monitoring techniques to take proper actions in order to prevent irreversible tissue damage. In addition, long-term adverse effects and the financial burden of immunosuppressor (IS) agents have resulted in a trend towards finding practical ways to replace these drugs with tolerance induction methods and to improve graft survival with molecular manipulation techniques. Therefore, finding molecules which play both diagnostic and therapeutic roles would be of great interest in this field.

The role of antiapoptotic molecules has already been recognized as both safe biomarkers in diagnosis of certain malignancies and potential targets in cancer therapy; however, manipulating antiapoptotic agents to improve graft survival is a novel topic to study in transplantation. There are also some efforts to produce IS drugs in combination with antiapoptotic agents in order to reduce these drugs toxic effects; for instance, Wu et al. demonstrated that JP-3-110 induces significantly less activated caspase-3 and apoptotic death of transplanted islet cells than mycophenolic acid, while exerting the same immunosuppressive effect [1]. Therefore, we tried to review the investigations exploring survivin's impact on allograft with the aim of bringing about innovative ideas for future experiments.

## 2. Survivin

Survivin was first described in 1997 by Ambrosini et al. as an antiapoptotic molecule involved in fetal development which is overexpressed in cancerous tissues. This small protein of 16.5 kDa is a member of inhibitor of apoptosis protein (IAP) family, responsible for apoptosis inhibition and cell cycle regulation [2]. The BIRC5 gene, encoding survivin is located on the long arm of chromosome 17 and its sequence is complementary to the effector cell protease receptor-1 (EPR-1) gene in reverse direction [3]. Transcription of survivin is directly regulated by suppressor molecule p53, given that accumulation of p53 in cell results in survivin depletion and apoptosis induction [4].

**Figure 1:**
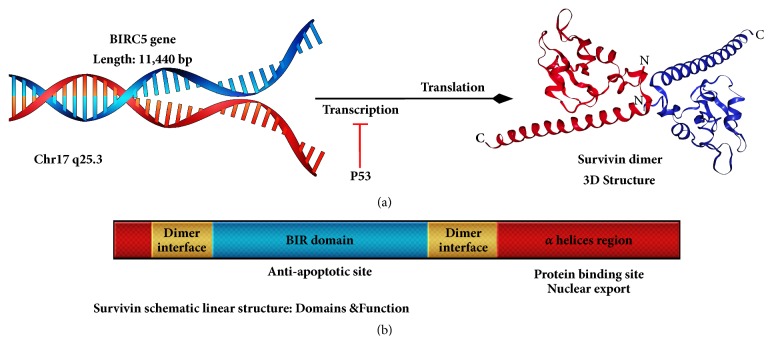
**Survivin gene and protein structure.** (a) The position of the gene and the relevant protein product with 3D dimer structure (base on PDB accession No.1e31) are shown. p53 protein plays an inhibitory role in the gene transcription. (b) The linear schematic structure of the survivin protein is shown along with the domains and roles of each. A BIR domain with antiapoptotic activity and an extended c-terminal alpha-helix region involved in protein interactions and nuclear exportation.

A specific feature of IAP family is the Baculoviral IAP Repeat (BIR) domain with conserved residues rendering a death-preventing activity to N-terminal by direct inhibition of caspase proteins. Survivin has a single BIR domain, which discriminates it from other IAPs. In addition, it has been found that the interaction between two hydrophobic positions in survivin structure forms a bow tie-shaped dimer in protein product [5] (Figure 1).

Zwerts et al. showed that lack of survivin causes mice to die at the early stages of embryogenesis due to the abnormal generation of heart. These mice also suffered from diffuse hemorrhage and neural tube defects [6].

After birth, survivin plays a significant role in viability and appropriate function of normal tissues, particularly those with high regeneration rates such as bone marrow [7], colonic mucosa [8], keratinocytes [9], thymus [10], liver [11], and endometrium [12].

Survivin overexpression in cancer has made it an appealing topic in molecular research because it has been shown that survivin overexpression is associated with high grade tumors and poor prognosis in a range of malignancies. Moreover, a link has been drawn between certain survivin gene polymorphisms (e.g., -31G/C) and susceptibility for cancer, as well as responsiveness to the chemotherapy [13]. In addition to the antiapoptotic role of survivin in oncogenesis, it has been shown that hypoxic condition of tumor environment could induce survivin expression in tumor cells which upregulates vascular endothelial growth factor (VEGF) synthesis and secretion leading to tumor angiogenesis [14]. Altieri has reported shortened survival, rapid disease progression, accelerated rates of recurrences, and increased resistance to chemotherapy in cancer patients with excessive expression of survivin; hence it could be an attractive target in molecular cancer therapy; there are also some suggestions to introduce it as a rapid prognostic and diagnostic biomarker in clinic [15].

Apoptosis can occur in various types of tissue injury such as ischemia, inflammation, drugs cytotoxicity, and infections [16], most of which can occur as a result of organ transplantation. Therefore, it might be useful to focus more on apoptotic and antiapoptotic pathways which affect allograft outcome and manipulate the involved molecules in order to prevent cell death.

## 3. Survivin in Transplantation

Contrary to cancer, tissue survival and apoptosis inhibition are considerable advantages in transplantation. To overcome ischemia reperfusion injury (IRI) and inflammation-induced tissue damages, has always been desired by clinicians; therefore, any means which makes the cells resistant to apoptosis would be welcomed in this area.

### 3.1. Survivin in Promoting Tissue Repair

Since kidney is the most transplanted organ, molecular studies are mainly directed to the renal transplantation. According to an investigation of Lechler and colleagues, survivin is expressed in adult renal epithelial cells, particularly in proximal tubules, and plays an essential role in tubular maintenance. They have even warned about targeting this molecule in cancer therapy because of its harmful effects on tubular integrity [17]. Musiał et al. studying chronic kidney disease (CKD) in children have observed increased amounts of survivin in patients' urine samples which was not in concordance with its serum level, so they suggested that this molecule is expressed in renal tissue probably as a protective factor against progressive damage and recommended survivin as a diagnostic maker in CKD follow-up; however, it could be presumed that survivin has been released and shed from tubules into the urine flow because of tissue destruction as a consequence of CKD pathogenesis [18].

Chen et al. demonstrated a protective role for survivin in mouse model of acute kidney injury (AKI). They compared ischemia reperfusion injury between the selective renal proximal tubule survivin knockout mice and a control group. The study showed a significant upregulation of survivin expression within 48 hours of IRI insult in control mice lasting for six days which was absent in knockout group; in addition, a progressive tubular damage was evident after two weeks of follow-up in the knockout mice comparing to the control group which exhibited only minimal injuries; therefore, they attributed delayed recovery and more tissue damage to the survivin deficiency in knockout mice [19]. Such a protective role was also demonstrated in the transgenic mice with 50% expression of survivin; after 7 days of folic acid-induced acute renal failure (ARF), significantly elevated levels of serum creatinine and increased tissue apoptosis were detected in transgenic group comparing to the normal mice. Besides, it was shown that survivin gene delivery to the deficient mice can restore the ability to avert tubular necrosis caused by ARF [20]. On the other hand, Lechler et al. found neither survivin gene upregulation nor protein overexpression in segmental renal infarction model; however, they observed continuously increasing signs of tissue proliferation 1, 3, and 7 days after ischemia induction [17].

Survivin involvement in tissue repair has also been investigated in other tissues. One such study used mesenchymal stem cells (MSCs) expressing survivin in mouse model of cardiac infarction. MSCs were infected with lentiviral vectors carrying green fluorescent protein (GFP) together with survivin genes (full-length mouse survivin cDNA without termination codon was amplified and together with GFP gene was inserted into the plasmid used for construction of recombinant lentiviral expression vector). After inducing heart permanent hypoxia, Fan and colleagues applied intramyocardial injection of phosphate-buffered saline (PBS), MSC-GFP, and MSC-GFP-SURVIVIN to three groups of mice. Cell survival was assessed one and four weeks later; accordingly, more cell viability, upregulated expression of VEGF, increased capillary density, reduced infarct size, less collagen deposition, and improved cardiac function were observed in MSC-GFP-SURVIVIN recipients compared to the other groups [21]. We could also point to a study about survivin overexpression in stroke model of rats. Self mesenchymal stem cells were infected by survivin/GFP-carrying lentiviral vectors and then were injected intravenously to the rats. The rats receiving survivin/GFP-MSCs showed significantly better survival rates after two hours of middle cerebral artery occlusion comparing to the GFP-MSCs recipient group. Mentioned groups of MSCs were also cultured in hypoxic condition and VEGF as well as basic fibroblast growth factor (bFGF) amounts were measured in culture supernatant. In vitro study displayed more VEGF and bFGF secretion from survivin/GFP-MCSs. Moreover, these mediators expression was shown to be increased in the brain tissue of the survivin/GFP-MCSs recipients, indicating the positive role of survivin in tissue repair after brain stroke [22]. In another study Qi Yuzeng et al. injected survivin-MSCs intravenously to the mice subsequent to the 24 hours of renal ischemia reperfusion. Seven days later they evaluated renal function by measuring serum creatinine and blood urine nitrogen (BUN) levels and showed that survivin-MSCs recipients had BUN and creatinine levels close to the sham group; therefore, it was concluded that survivin-MSCs could attenuate renal ischemia reperfusion injury. Transplanted MSCs stability was also assessed in kidney tissue and it was demonstrated that hepatocyte growth factor (HGF) and bFGF proteins' expression and number of MSCs in survivin-MSCs recipients' renal tissue were significantly more than mock-MSCs recipients [23].

### 3.2. Survivin in Promoting Graft Survival

Regarding antiapoptotic effects of survivin, there have been some interventional experiments to apply such a property in transplantation for the purpose of promoting engraftment and allograft survival. In an experiment by Dohi et al. survivin expression in mice transgenic (full-length mouse survivin complementary DNA was cloned and microinjected into C57Bl/6 embryos) *β*-cells affected neither insulin release nor glucose levels; moreover, there was no difference between wild-type and transgenic mice in islets size and number. Nevertheless, the latter group was protected against staurosporine (STS)-induced cell death comparing to the other.

Besides, they transplanted wild-type and survivin-expressing islet cells (infected with a replication-deficient adenovirus encoding haemagglutinin-tagged wild-type survivin) to the streptozotocin (STZ)-induced diabetic mice. After one and two months of follow-up corrected hyperglycemia and appropriate glucose tolerance were observed in all transplanted diabetic mice receiving transgenic *β*-cells while there has not been any curative effect using normal cells. So they demonstrated survivin molecule's effectiveness in protecting tissue against STS toxicity and transplantation insults [24].

P.Cassis et al. have also generated mice with 50% of survivin gene expression (haplo-insufficient) and investigated different syngenic as well as allogenic renal transplant models. They reported three main findings: first, survivin haplo-insufficient mice would reject syngenic kidney grafts due to the sustained inflammation and deficient tissue repair after a period of ischemia; the second finding was that survivin gene delivery (using plasmid vectors) could improve tissue survival in both syngenic and allograft models; finally, in fully mismatch transplantation models survivin overexpression resulted in better graft survival and less chronic allograft nephropathy. Besides, considering the oncogenic potential of survivin, they commented that no malignancy was detected in overexpressing group during a long-term follow-up period [25].

Quillard et al. has shown that Notch2 gene silencing which regulates survivin gene expression could protect endothelial cells from TNF-induced apoptosis [26]; this finding suggests a protective role for survivin during acute vascular rejection episodes when there is a considerable rise of proinflammatory cytokines in allograft activating apoptotic pathways.

Furthermore, it has been shown that survivin inhibition in mouse and human bone marrow results in fewer MSC and clonogenic colony forming unit fibroblasts, whereas survivin overexpression in MSC promotes their proliferation; moreover, survivin inhibition in MSCs reduces their hematopoiesis-supporting capacity. According to the considerable role of MSCs in engraftment and maintenance of hematopoietic stem cell transplantation (HSCT), survivin-expressing MSCs seem to promote HSCT outcome [27]. In addition, erythrocytes have been demonstrated to be significantly dependent on survivin in their proliferation and maturation process, as heterozygous deletion of survivin in mice is accompanied by decreased number of enucleated erythrocytes and insufficient erythropoiesis while its complete deletion causes death [28]. Gurbuxani et al. also showed that survivin is definitely required for megakaryocytic and erythroid progenitors' development, although its expression diminishes in terminal stages of megakaryocytes' differentiation [7].

### 3.3. Concerns about Applying Survivin in Clinic

#### 3.3.1. Augmented Risk of Malignancy

In spite of positive effect of survivin overexpression on allograft survival, this antiapoptotic molecule's involvement in cancer arises some concerns about its use in clinic. Decision-making would get even more complicated considering the increased risk of posttransplant lymphoproliferative disorders due to the intensive immunosuppression.

A notable study in this regard was carried out by Michele Bernasconi et al. to investigate early molecular events leading to B-cell transformation after Epstein-Barr virus (EBV) infection. They used tonsils as the source of B lymphocytes. After induction of EBV infection, gene expression profile assay was performed using Affymetrix microarray. Three genes whose expression had been significantly elevated by EBV were cyclin-dependent kinase 1 (CDK1), cyclin B1 (CCNB1), and survivin [29]; this finding in addition to our knowledge about survivin implication in cancer indicates that its application for graft promotion should be cautiously studied to avoid inducing malignancies.

#### 3.3.2. T and B Lymphocytes Overactivation

Another concern about survivin overexpression is its role in T lymphocytes homeostasis. Zheng Xing et al. inactivated survivin gene in mice and performed an apoptosis assay on primary thymocytes and peripheral lymphocytes. The survivin-deficient mice at early stages of thymic development showed impaired pre T cell proliferation. Although later stages passed normally at thymus, peripheral blood T cells displayed immature phenotypes and reduced number. These findings propose an essential function for survivin at both early and late phases of T cells development [30]. Survivin contribution to T lymphocytes proliferation has also been described by Jianxun Song et al. as their study showed that OX40 signaling in T cells controls survivin expression; therefore, survivin transduction could rescue T cells proliferation in OX40-deficeint mice. Moreover, they demonstrated that survivin maintains T cell division and clonal expansion [31]. These findings call for a question: if survivin deficiency induces T cells insufficiency, could we expect an exaggerated T cell proliferation/activation by this molecule's overexpression? Regarding unfavorable role of allo-reactive T lymphocytes in transplantation as well as these cells substantial implication in recruiting and activating other components of immune system, the potential risk of allograft rejection should be considered in survivin overexpression.

B cells life cycle is also markedly affected by survivin. Miletic et al. studying mice bone marrow showed survivin high expression in proliferating CD43+B220+IgM−IgD− cells which is maintained through small pre-B cell stage but it was undetectable in immature B cells. Subsequent to mitotic stimulation as well as in germinal centers, survivin expression is restored significantly in mature B cells. In addition, the mice lacking survivin expression failed to develop sufficient B lymphocytes in BM. Although survivin seems not to be required for mature B cells maintenance, it is necessary for their proper function as it was demonstrated that IgM and IgG serum levels along with peritoneal B cell populations were reduced in survivin^L/L^CD21^Cre^ mice [32]. According to these findings indicating survivin role in B cells development and activity, the same concern exists about these molecules overexpression in transplantation as it might stimulate B lymphocytes proliferation and excessive antibodies secretion.

## 4. Conclusion

**Figure 2:**
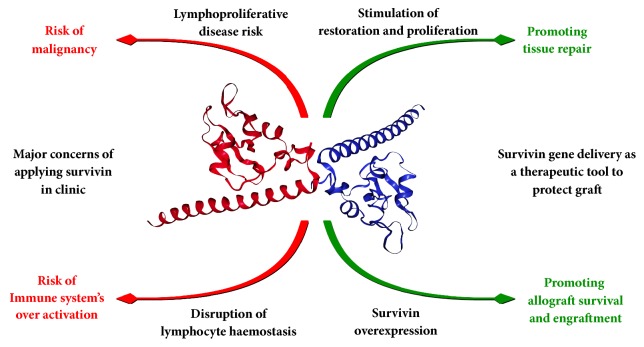
**An overview of the role of survivin in transplantation.** The effects of survivin on the transplantation are categorized in two general groups of the advantages and limitations.

In brief, to protect allograft from apoptosis induced by various insults such as ischemia reperfusion injury, toxic effect of IS drugs, infection, and sustained allo-responses leading to chronic rejection, it might be useful to apply synthetic antiapoptotic agents or induce overexpression of the biologic ones like survivin. Regarding the findings of gene deletion and overexpression studies, there is some evidence in favor of survivin's beneficial effect on tissue repair, ischemia resistance, and engraftment; nonetheless, the risk of developing malignancies and lymphocytes overactivation should be taken into account. To overcome these risks, it is suggested to apply tissue restricted survivin expression for a limited time such as reperfusion period posttransplant (Figure 2).
